# CpG and Interleukin-15 Synergize to Enhance IFN-**γ** Production by Activated CD8^+^ T Cells

**DOI:** 10.1155/2013/924023

**Published:** 2012-12-23

**Authors:** Dustin Cobb, Siqi Guo, Ronald B. Smeltz

**Affiliations:** ^1^Department of Microbiology and Immunology, Virginia Commonwealth University, P.O. Box 980678, Richmond, VA 23298, USA; ^2^Department of Microbiology, Old Dominion University, 4211 Monarch Way, Suite 300, Norfolk, VA 23508, USA

## Abstract

Interleukin-15 (IL-15) regulates the development and maintenance of memory CD8^+^ T cells. Paradoxically, we previously reported that IL-15 could enhance CD8^+^ T-cell responses to IL-12, a proinflammatory cytokine required for optimal priming of effector CD8^+^ T cells. To expand the physiological relevance of these findings, we tested IL-15 for its ability to enhance T-cell responses to bacterial CpG. Expectedly, CpG enhanced the production of IFN-*γ* by CD8^+^ T cells polyclonally activated with anti-CD3. However, addition of IL-15 to CpG-stimulated cultures led to a striking increase in IFN-*γ* production. The effect of CpG and IL-15 was also evident with CD8^+^ T cells recovered from mice infected with the parasite *Trypanosoma cruzi* (*T. cruzi*) and restimulated with antigen. The observed synergy between CpG and IL-15 occurred in an IL-12-dependent manner, and this effect could even be demonstrated in cocultures of activated CD8^+^ T cells and CD4^+^CD25^+^ regulatory T cells. Although IFN-*γ* was not essential for CpG-induced IL-12, the ability of CpG and IL-15 to act on CD8^+^ T cells required expression of the IFN-*γ*-inducible transcription factor T-bet. These data have important implications for development of vaccines and design of therapies to boost CD8^+^ T-cell responses to infectious agents and tumors.

## 1. Introduction

Interleukin-15 (IL-15) is a member of the common *γ* chain cytokine family that is important for development and maintenance of memory CD8^+^ T cells [1, 2]. IL-15 has shown promise in its ability to enhance vaccine-mediated immunity against pathogens. For example, vaccination against the intracellular protozoan parasite *Trypanosoma cruzi *(*T. cruzi*), the causative agent of human Chagas disease, was improved when combined with a plasmid encoding IL-15 [3]. IL-15 can also promote the effector functions of CD8^+^ T cells [4]. For instance, we previously demonstrated that IL-15 enhances IFN-*γ* production by human CD8^+^ T cells by increasing T cell responsiveness to IL-12 [5]. Others have demonstrated that adoptive immunotherapy of solid tumors was enhanced by inducing a lymphopenic environment in the tumor-bearing host prior to adoptive transfer of T cells, and that IL-15-mediated lymphopenia-induced proliferation (as well as proinflammatory cytokines released in response to total body irradiation) was an important component of effective therapy [6].

CpG motifs are unmethylated dinucleotides that are present in bacterial DNA, and they were previously discovered to possess powerful immunostimulatory properties [7, 8]. Binding of CpG to Toll-like receptor 9 (TLR-9) on antigen-presenting cells (APCs) induces APC maturation through up-regulation of MHC class II, and the costimulatory molecules CD40 and CD86 [9, 10]. CpG can also stimulate production of proinflammatory cytokines, particularly IL-12, which promotes T-cell priming and differentiation [11]. Thus, CpG has the potential to enhance both innate and adaptive immunity and represent a powerful agent that can be used as an adjuvant for vaccine-induced immunity. Indeed, the immunostimulatory properties of bacterial CpG have been recapitulated by the use of synthetic oligodeoxynucleotides (ODNs). For example, synthetic CpG have been shown to increase both natural and vaccine-induced immune responses to *T. cruzi *[12].

Based on our previous studies with IL-15, and to examine the efficacy of IL-15 in a clinically relevant manner, we sought to determine as a proof-of-concept if IL-15 could synergize with CpG to enhance CD8^+^ T-cell responses. We report that the combination of IL-15 and CpG led to a significant increase in IFN-*γ* production by CD8^+^ T cells, including the enhancement of IFN-*γ* production by *T. cruzi*-specific CD8^+^ T cells in an antigen-specific manner. Mechanistically, the observed synergy between IL-15 and CpG was critically dependent upon APC-derived IL-12. The potency of the combined effect of CpG-induced IL-12 and IL-15 was also evident in cocultures of CD8^+^ T cells and naturally occurring (i.e., thymus-derived), CD4^+^CD25^+^ regulatory T cells (nTreg), which are well known for their potent ability to suppress T-cell activation. Impressively, both proliferation and IFN-*γ* production were markedly enhanced by these cytokines even in the presence of high numbers of nTreg. Although IFN-*γ* was not essential for CpG-induced IL-12, the ability of CpG and IL-15 to act on CD8^+^ T cells required expression of the IFN-*γ*-inducible transcription factor T-bet. These results have important implications for future development of preventative vaccines that combine the potency of TLR agonists such as CpG with cytokines known to promote long-term memory.

## 2. Materials and Methods

### 2.1. Mice and Infections

Age-matched female C57BL/6, *Tbx21* ^−/−^, B6.129 *IL12p35* ^−/−^, and *Ifng* ^−/−^ mice were obtained from The Jackson Laboratory and were used between six and eight weeks of age. Mice were housed in an Association for Assessment and Accreditation of Laboratory Animal Care (AAALAC)- accredited facility under pathogen-free conditions and used in accordance with an Institutional Animal Care and Use Committee- (IACUC)- approved protocol. For infections, mice were injected intraperitoneally with 1 × 10^6^ tissue-culture-derived *T. cruzi* trypomastigotes (CL strain).

### 2.2. Reagents and Cell Purifications

Recombinant human IL-15 was purchased from R&D Systems and used at 1 ng/mL, 10 ng/mL, or 100 ng/mL. CpG ODN 231627 was purchased from TIB MolBiol. For antigen-specific *ex vivo* recall responses to *T. cruzi*, Tskb20 peptide was used. Tskb20 peptide (ANYKFTLV) was synthesized by and purchased from Abgent (San Diego, CA). For *in vitro* neutralization of IFN-*γ* and IL-12, LEAF-purified anti-IFN-*γ* (clone XMG1.2, Biolegend) and anti-IL-12p40 (clone C17.8, Biolegend) antibodies were used at a concentration of 5 *μ*g/mL. CD8^+^ T cells were isolated from naïve and *T. cruzi*-infected mice by positive selection using CD8 Microbeads (MACS, Miltenyi) and purity was greater than 95%. Thy1.2^−^ splenocytes were isolated by depleting Thy1.2^+^ cells from naïve or *T. cruzi*-infected mice using CD90.2 Microbeads and LD columns (MACS, Miltenyi) and purities were greater than 98%. CD8^+^ T cells were activated with soluble anti-CD3 (0.1 *μ*g/mL, 145-2C11). CD4^+^CD25^+^ nTreg were prepared from pooled lymph nodes of naïve mice using Treg isolation kits (Miltenyi Biotec). Purity of nTreg populations (CD4^+^CD25^+^) was routinely checked by FACS and greater than 93% and purified nTreg expressed Foxp3 as determined by intracellular FACS.

### 2.3. *In Vitro* and *Ex Vivo* Cell Cultures

2 × 10^5^ CD8^+^ T cells from naïve mice were cocultured with 2 × 10^6^ live Thy1.2-depleted splenocytes in complete RPMI 1640 (10% FBS, 10 mM Hepes, 1 IU/mL penicillin and 100 *μ*g/mL streptomycin, 2 mM L-glutamine, 1 × 10^−5^ M 2-*β*-mercaptoethanol). Cells were stimulated with 0.1 *μ*g/mL of LEAF-purified anti-CD3 in the presence of 0.5 *μ*M CpG and recombinant human IL-15 for 72 hours. For antigen-specific responses, 2 × 10^6^ splenocytes from *T. cruzi*-infected mice were cultured with Tskb20 peptide (1 ng/mL) for 72 hours.

### 2.4. CFSE Labeling

Subsequent to column purification, CD8^+^ T cells were labeled with CFSE by incubating T cells at 100 × 10^6^ cells/mL in 5 *μ*M of CFSE/HBSS for 5 minutes at room temperature. Labeling was quenched by addition of an equal volume of fetal bovine serum. Cells were washed and resuspended in complete RPMI.

### 2.5. Coculture Assay with Treg

Cocultures of CFSE-labeled CD8^+^ T cells, APC, and Treg were established by adding 2.5 × 10^5^ CD8^+^ T cells, 1 × 10^6^ APC, and decreasing numbers of Treg (starting with a 1 : 1 suppressor : responder ratio) per mL of complete RPMI to wells of 48-well plates. Low-endotoxin/azide-free anti-CD3 antibody (LEAF, 2C11, Biolegend) was used at 0.5 *μ*g/mL. In addition, recombinant human IL-15 (R&D Systems) and recombinant mouse IL-12 (R&D Systems) were used at 1 ng/mL and 0.1 ng/mL, respectively. After 72 hours, cells were harvested and supernatants stored frozen at −80 C until use. Cells were stained with anti-CD8 allophycocyanin (Biolegend), and 30,000 live events were analyzed on a Beckman-Coulter FC500 instrument. Quantification of cell division was determined by gating on either CFSE^+^ cells or CD8^+^ T cells and determining the frequency of cells in each gate/CFSE peak. Supernatants were tested for IFN-*γ* as described below.

### 2.6. Measurement of Cytokines

Cell culture supernatants were collected after 72 hours, or in some cases after 24 hours, and analyzed for the presence of IFN-*γ* and IL-12p40 using Biolegend's ELISA MAX Standard Sets according to their recommendations.

### 2.7. Statistical Analysis

Data were analyzed using one-way ANOVA, Tukey multiple comparison procedures (SigmaPlot 11.0, Systat Software, Inc.). A *P* value <0.05 was considered significant. Data are represented as means ± SD of experimental groups.

## 3. Results

### 3.1. IL-15 and CpG Synergize to Enhance IFN-*γ* Production by CD8^+^ T Cells

**Figure 1:**
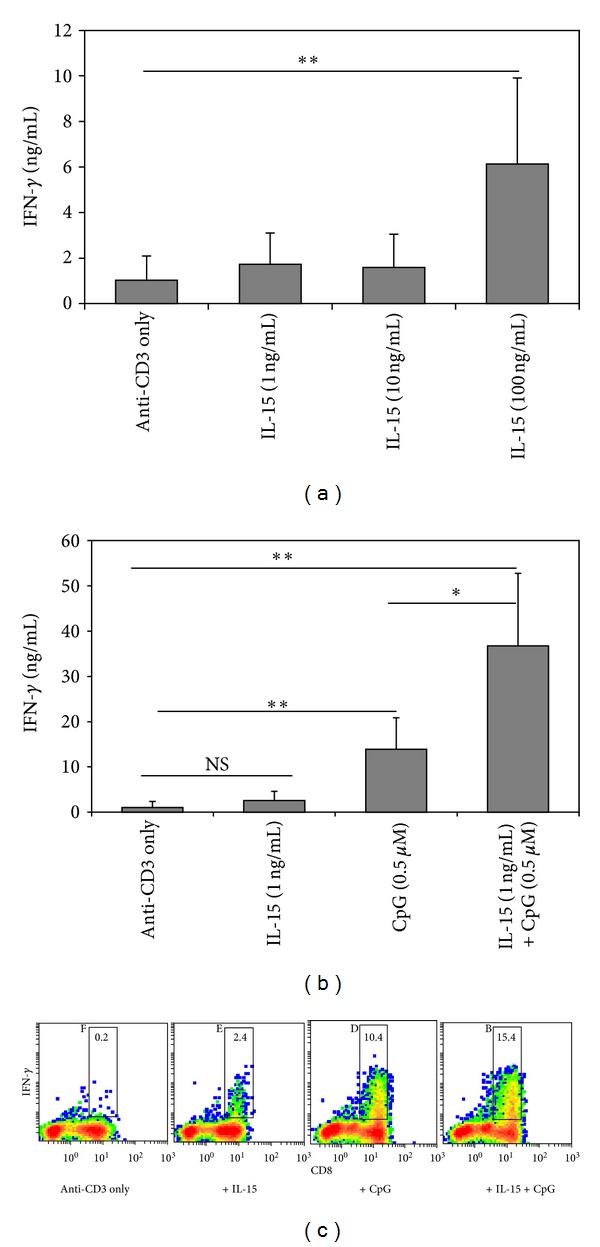
The synergistic effects of IL-15 and CpG enhance CD8^+^ T cell IFN-*γ* production. (a) Naïve CD8^+^ T cells were cocultured with live Thy1.2-depleted splenocytes and anti-CD3 (0.1 *μ*g/mL) with increasing concentrations of recombinant human IL-15. After 72 hours culture supernatants were harvested and tested by ELISA for IFN-*γ*. (b) CD8^+^ T cells and Thy1.2-depleted splenocytes were cocultured with anti-CD3 for 72 hours in the presence of IL-15 (1 ng/mL), CpG (0.5 *μ*M), or IL-15+CpG. Cell culture supernatants were then harvested and IFN-*γ* levels were determined by ELISA. (c) Intracellular cytokine staining and flow cytometric analysis comparing CD8 expression (*x*-axis) versus IFN-*γ* production (*y*-axis) following 72 hour culture with IL-15+CpG. Numbers represent the percentage of CD8^+^ T cells that are IFN-*γ* ^+^. Results are representative of three independent experiments and are expressed as mean ± SD. **P < 0.005; *P < 0.05; NS: not significant.

Previous studies have demonstrated the importance of IL-15 in regulating the development and function of memory CD8^+^ T cells. Additionally, we reported that IL-15 could enhance human T-cell responses to IL-12. To assess the physiological relevance of this effect, we sought to determine whether IL-15 and CpG could augment IFN-*γ* production by TCR-activated CD8^+^ T cells. At low doses (1–10 ng/mL), IL-15 alone had very little impact on IFN-*γ* production (Figure 1(a)). However, when 100 ng/mL of IL-15 was used, there was a significant increase in the level of IFN-*γ* production (Figure 1(a)). Thus, to minimize direct effects of IL-15 on cytokine production, we used 1 ng/mL of IL-15 for all subsequent experiments. We next evaluated the combined effects of IL-15 and CpG on IFN-*γ* production by CD8^+^ T cells. As expected, addition of IL-15 to anti-CD3-stimulated CD8^+^ T cells did not increase in IFN-*γ* production. However, addition of CpG led to a significant increase in TCR-induced IFN-*γ* production compared to anti-CD3 stimulation only (Figure 1(b)). Surprisingly, the addition of both IL-15 and CpG to CD8^+^ T-cell cultures stimulated with anti-CD3 resulted in strong enhancement of IFN-*γ* production (Figure 1(b)). Importantly, the increase in IFN-*γ* production represented a synergistic effect. To demonstrate that the effect of IL-15 and CpG was on CD8^+^ T cells only, intracellular cytokine staining/FACS were performed. In agreement with the above results, the percentage of CD8^+^ T cells producing IFN-*γ* was significantly increased following treatment with both IL-15 and CpG (Figure 1(c)). In contrast, CD8-negative cells (which include CD4^+^T cells and NK1.1 cells) failed to show a similar increase (Figure 1(c)). These results are further supported by the fact that less than 1% of these cells were present in CD8^+^ T-cell cultures and did not produce significant amounts of IFN-*γ* following culture with IL-15 and CpG. Furthermore, Thy1.2-depleted (T-cell-depleted) splenocytes cultured in the presence of IL-15+CpG produced little IFN-*γ* (<0.1 ng, not shown), indicating that CD8^+^ T cells are the main source of IFN-*γ* in these cultures. These results demonstrate that IL-15 can enhance the ability of CpG to promote IFN-*γ* production by TCR-activated CD8^+^ T cells, without directly inducing IFN-*γ*. These data are consistent with a report in which the combination of IL-15 and CpG-enhanced CD69 expression on human T cells [6].

### 3.2. IL-15 and CpG Can Synergize to Increase the T-Cell IFN-*γ* Response to the Intracellular Parasite *T. cruzi* in an Antigen-Specific Manner

**Figure 2:**
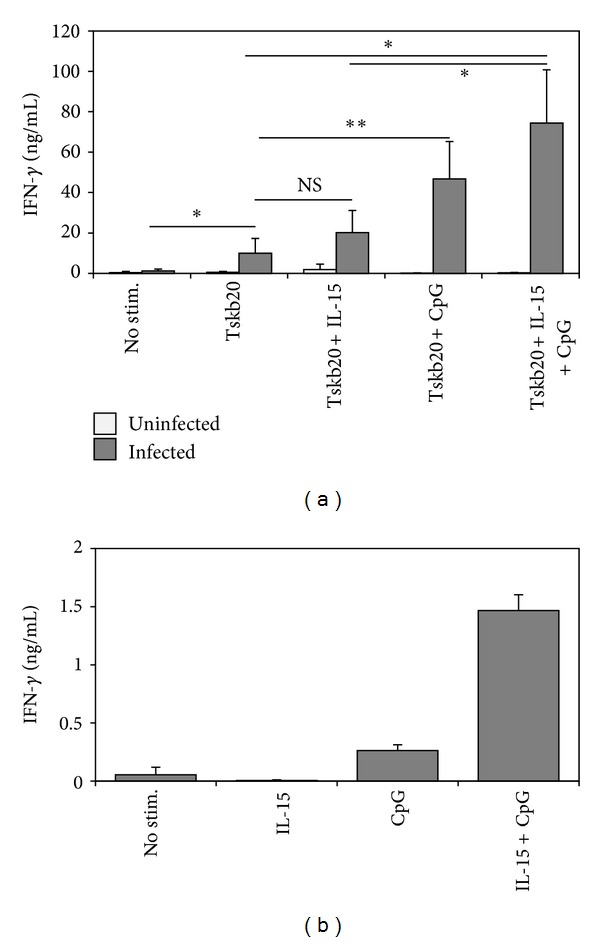
The synergistic effects of IL-15 and CpG enhance *T. cruzi*-specific IFN-*γ* production by CD8^+^ T cells. C57BL/6 mice were infected with *Trypanosoma cruzi*. On day 9 postinfection, mice were euthanized and spleens harvested. (a) Splenocytes were cultured for 72 hours with Tskb20 peptide (1 ng/mL) to induce an antigen-specific CD8^+^ T cell response. Culture supernatants were tested by ELISA to determine the levels of IFN-*γ* production. (b) Purified CD8^+^ T cells were cultured in the presence of IL-15, CpG, or IL-15+CpG for 72 hours. Culture supernatants were tested by ELISA for IFN-*γ*. **P < 0.005; *P = 0.05; NS: not significant. Results are representative of at least three independent experiments with two mice per group and are expressed as mean ± SD.

Because of the potent synergy between CpG and IL-15 in enhancing IFN-*γ* production by T cells polyclonally stimulated with anti-CD3, we next wanted to determine if this effect could be extended to antigen-specific CD8^+^ T cells. To test this, we utilized a murine model of *T. cruzi* infection. Infection with the intracellular protozoan parasite *T. cruzi* elicits a strong CD8^+^ T-cell-mediated IFN-*γ* response that is necessary for host protection. Previous studies have also shown that successful vaccine-induced immunity against *T. cruzi* induces a strong CD8^+^ T-cell and IFN-*γ* response [13–16]. Hence, mice were infected with *T. cruzi* and splenocytes harvested 9 days postinfection (day 9 p.i.). This time point was chosen because we can detect *T. cruzi*-specific CD8^+^ T cells in infected mice by FACS on this day [17], and it has been demonstrated to peak at approximately 10–12 d.p.i. [18]. Splenocytes from uninfected and *T. cruzi*-infected mice were subsequently restimulated with Tskb20, a peptide previously identified as an immunodominant epitope of the CD8^+^ T-cell response to *T. cruzi*. Addition of IL-15 to Tskb20 peptide-stimulated splenocyte cultures did not significantly increase IFN-*γ* production. However, similar to its effects on polyclonally activated CD8^+^ T cells from uninfected mice, CpG induced a significant increase in Tskb20 antigen-specific IFN-*γ*. Addition of IL-15 to CpG led to a further increase in IFN-*γ* production by Tskb20-specific CD8^+^ T cells, albeit not as significant as that observed with naïve T cells (Figure 2(a)). Since these T cells were derived from infected mice and highly activated, we wanted to determine whether IL-15 and CpG could drive IFN-*γ* production in the absence of TCR stimulation. To test this, CD8^+^ T cells were purified from *T. cruzi*-infected mice and cultured in the presence of IL-15, CpG, or IL-15+CpG. Although the levels of IFN-*γ* production were lower without TCR stimulation, the synergistic effects of IL-15 and CpG were still evident (Figure 2(b)). Thus, comparable to anti-CD3-stimulated CD8^+^ T cells, IL-15 and CpG can synergize to promote antigen-specific IFN-*γ* production by CD8^+^ T cells. Of note, IFN-*γ* levels from Thy1.2-depleted (T cell-depleted) splenocytes cultured in the presence of IL-15/CpG were minimal (<1 ng/mL, not shown), indicating a minor contribution of APC to IFN-*γ* production.

### 3.3. The Enhancement of T-Cell IFN-*γ* Production by IL-15 and CpG Requires IL-12 Production by Antigen-Presenting Cells

**Figure 3:**
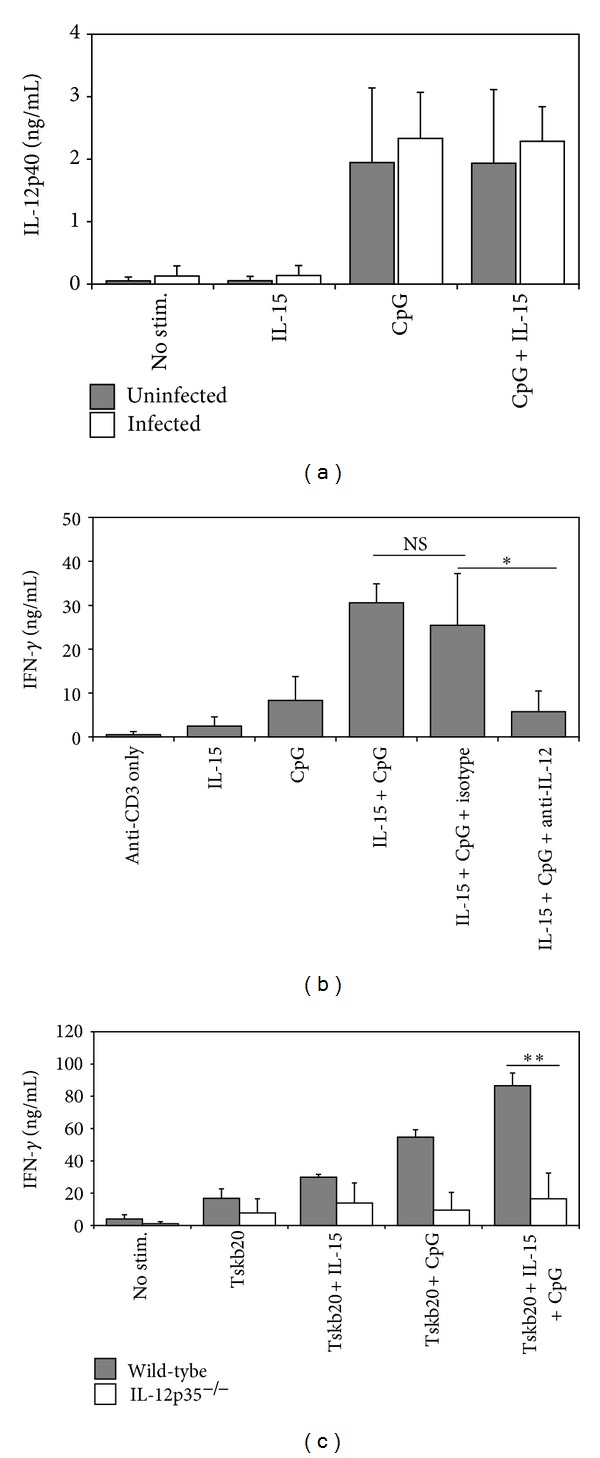
IL-12 is required for CpG+IL-15-mediated enhancement of IFN-*γ* production. (a) Splenocytes were harvested from the spleens of uninfected (filled bars) or *T. cruzi*-infected wild-type (open bars) mice on day 9 p.i. Thy1.2-depleted splenocytes were then cultured in the presence of IL-15, CpG, or both IL-15 and CpG. After 72 hours, culture supernatants were harvested and tested for IL-12p40 by ELISA. (b) CD8^+^ T cells and live Thy1.2-depleted APC were cocultured with anti-CD3 for 72 hours in the presence of IL-15, CpG, IL-15+CpG, IL-15+CpG+isotype antibody, or IL-15+CpG+anti-IL-12p40 antibody. Cell culture supernatants were then harvested and IFN-*γ* levels were determined by ELISA. (c) Splenocytes from wild-type (filled bars) or *IL-12p35* ^−/−^ (open bars) mice infected with *T. cruzi* were cultured for 72 hours with Tskb20 peptide. **P < 0.005; *P = 0.05; NS: not significant. Results are representative of three to four independent experiments with at least two mice per group and expressed as mean ± SD.

Previously it was shown that IL-15 is essential for CpG-induced activation of dendritic cells, including the production of IL-12 [19]. Therefore, we sought to determine whether the synergistic effect of IL-15 and CpG was a consequence of increased IL-12 production by APC. To test this, APC cultures were prepared by depletion of Thy1.2^+^ cells from splenocytes obtained from either uninfected or *T. cruzi*-infected mice, and subsequently cultured with IL-15, CpG, or IL-15 plus CpG. Indeed, stimulation of APC with CpG resulted in a significant increase in IL-12p40 production. Surprisingly, IL-15 alone had no significant effect on IL-12p40 production. Furthermore, the combination of IL-15 and CpG did not further increase the levels of IL-12p40 over that of CpG alone (Figure 3(a)). Thus, these data show that although CpG induces IL-12 secretion, synergy between IL-15 and CpG is not the result of an IL-15-mediated increase in IL-12 production.

Although CpG are known to stimulate IL-12 production, it was important from a mechanistic standpoint to determine if CpG-induced IL-12p40 was in fact required for IL-15+CpG-induced IFN-*γ* production by CD8^+^ T cells. To address this issue, we first neutralized IL-12p40 in CD8^+^T cell cultures stimulated with anti-CD3, CpG, and/or IL-15. Clearly, neutralization of IL-12p40 led to a dramatic reduction in CpG+IL-15-induced IFN-*γ* production (Figure 3(b)). Since both IL-12 and IL-23 share the IL-12p40 subunit, we wanted to confirm the importance of APC-derived IL-12 in CpG+IL-15-mediated effects on *T. cruzi*-specific CD8^+^ T cells. To do so, we used APCs purified from *IL12p35* ^−/−^ mice (which specifically lack IL-12). Similar to results shown in Figure 3(b), splenocytes from *T. cruzi*-infected *IL12p35* ^−/−^ mice failed to exhibit an increase in antigen-specific IFN-*γ* production following IL-15 and CpG treatment (Figure 3(c)). Furthermore, experiments revealed that addition of recombinant IL-12 recapitulated the effect of CpG (data not shown). Thus, the ability of IL-15 and CpG to synergize and enhance IFN-*γ* production by *T. cruzi*-specific CD8^+^ T cells is driven by CpG-induced IL-12 production by APC.

### 3.4. Synergy between IL-12 and IL-15 Overcomes Potent Suppression of CD8^+^ T-Cell Responses by CD4^+^CD25^+^ Regulatory T Cells

**Figure 4:**
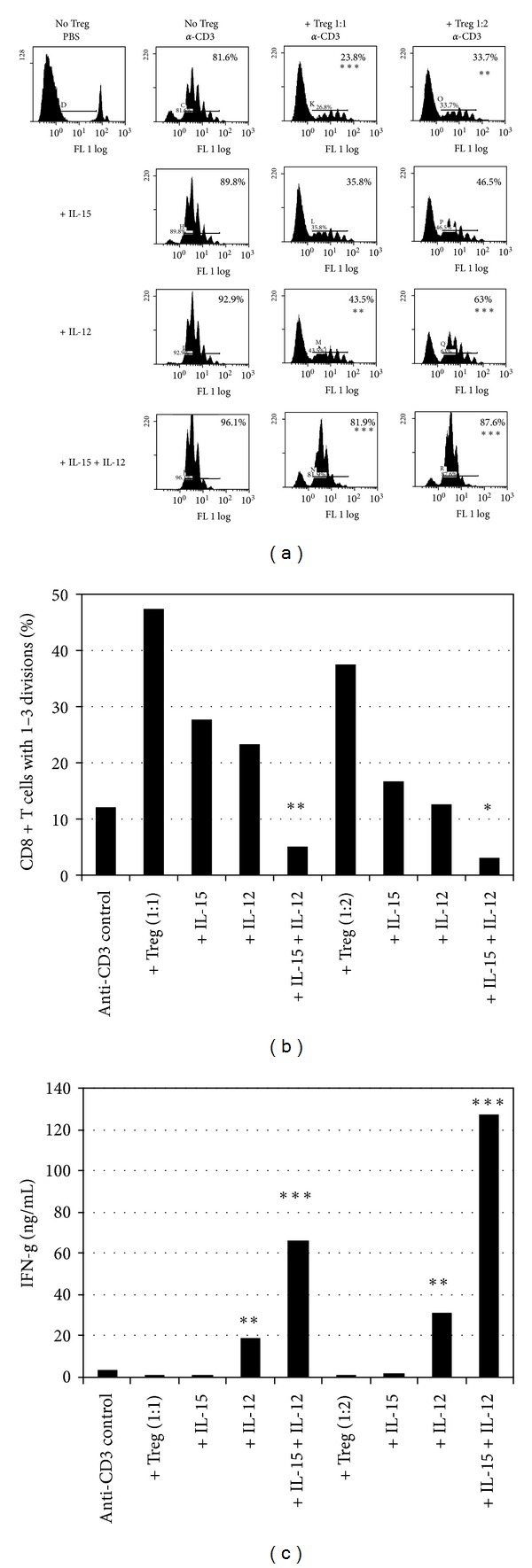
Synergy between IL-12 and IL-15 overcomes potent suppression of CD8^+^ T-cell responses by nTreg. (a) CFSE-labeled CD8^+^ T cells, APC, and anti-CD3 were cultured in the absence or presence of purified nTreg at a 1 : 1 or 1 : 2 Treg : CD8 T-cell ratio, along with either IL-12, IL-15, or IL-12+IL-15. After 72 hours, cells were analyzed for CFSE dilution by FACS. 30,000 live events were collected, percentages reflect the number of CFSE^lo^, or dividing CD8^+^ T cells (***P < 0.001, **P < 0.01). (b) 30,000 CD8^+^ T cells were analyzed to determine the frequency of CD8^+^ T cells that had divided either 1–3 or >4 times. Numbers reflect the percentage of cells accumulated in either group. *P* values for cytokine-treated wells are relative to Treg suppression controls (no cytokines). **P < 0.01 for IL-12+IL-15 relative to IL-12; *P < 0.05 for IL-12+IL-15 relative to IL-12. In (c), supernatants were harvested from cocultures and analyzed for IFN-*γ* by ELISA (***P < 0.001, **P < 0.01). Results of one experiment are shown, asterisks represent the statistical significance of each group from at least 4 experiments.

Since IL-12 was the primary mediator of the CpG effect, we next wanted to determine if IL-12 could synergize with IL-15 to enhance IFN-*γ* production when CD8^+^ T cells were activated in the presence of CD4^+^CD25^+^Foxp3-expressing regulatory T cells (nTreg), a T-cell subset well known for its ability to suppress both CD8^+^ and CD4^+^ T cell functions. Thus, we performed coculture experiments consisting of CFSE-labeled CD8^+^ T cells, APC, anti-CD3, and purified nTreg at either a 1 : 1 or 1 : 2 suppressor : responder ratio. In the absence of nTreg, a majority of anti-CD3-stimulated CD8^+^ T cells were CFSE^dim⁡^ after 72 hours (Figure 4(a), 79.7%). In contrast, coculture with nTreg suppressed CD8^+^ T-cell proliferation in a concentration-dependent fashion, reducing the frequency of CFSE^dim⁡^ cells (Figure 4(a), 40.9% and 47% for 1 : 1 and 1 : 2 ratios, *P* < 0.001 and *P* < 0.01 resp.). In addition to reduced proliferation, IFN-*γ* production was suppressed to undetectable levels at both ratios tested (*P* < 0.001 for 1 : 1, *P* < 0.01 for 1 : 2, Figure 4(c)). However, addition of IL-12 to cocultures led to a significant increase in CD8^+^ T-cell proliferation (Figure 4(a), 70% and 80.8% for 1 : 1 and 1 : 2 ratios, *P* < 0.01 and *P* < 0.001 resp.). Furthermore, exogenous IL-12 induced significant IFN-*γ* production (Figure 4(c), *P* < 0.01 and *P* < 0.001 for 1 : 1 and 1 : 2 ratios, resp.). In contrast to IL-12, IL-15 had a modest enhancing effect on proliferation, but did not reach statistical significance (Figure 4(a)). Similarly, IL-15 alone had no significant effect on IFN-*γ* production. Despite these modest effects, however, addition of IL-15 to cocultures containing IL-12 led to a striking increase in both CD8^+^ T cell proliferation (Figure 4(a), *P* < 0.001 for both 1 : 1 and 1 : 2 ratios) and IFN-*γ* production (Figure 4(c), *P* < 0.001, for both 1 : 1 and 1 : 2 ratios).

In addition to changes in the frequency of CSFE^dim⁡^ CD8^+^ T cells (Figure 4(a)), differences existed in the number of cell divisions among CFSE^dim⁡^ cells. Thus, we performed FACS analysis by gating on live CD8^+^ T cells and determined the frequency of cells that had either 1–3 or >4 cell divisions. The significance of this number is that at least 3 cell divisions are required before CD8^+^ T cells express the chemokine receptor CXCR3, an important phenotypic marker of effector T cells (unpublished data). The advantage of gating on CD8^+^ T cells is that it allows us to account for cells that had lost their CFSE due to extensive proliferation (>6 cell divisions). A majority of CD8^+^ T cells cultured with anti-CD3 divided four or more times (Figure 4(b), 87% of CD8^+^ T cells), while only 12% of CD8^+^ T cells divided 1–3 times. However, coculture of CD8^+^ T cells with nTreg led to a significant increase in CD8^+^ T cells that had divided only 1–3 times (47.3% and 37.4% for 1 : 1 and 1 : 2 ratios, resp., *P* < 0.001). When IL-12 was added to cocultures, however, the number of cells with 1–3 divisions was reduced to 23.2% and 12.5% (1 : 1 and 1 : 2 ratios, resp.) as more CD8^+^ T cells divided >4 times (Figure 4(b), *P* < 0.001). Surprisingly, IL-15 was equivalent to IL-12 for its ability to increase cell division among CFSE^dim⁡^ cells (*P* < 0.01 at 1 : 1, *P* < 0.001 for 1 : 2 ratio). Importantly, the addition of IL-15 to cocultures with IL-12 caused a significant increase in CD8^+^ T cell division, with only 5% and 3% of CD8^+^ T cells with 1–3 cell divisions (Figure 4(b), *P* < 0.001, *P* < 0.01 for difference between IL-12 and IL-12+IL-15 at 1 : 1 ratio, *P* < 0.05 for difference between IL-12 and IL-12+IL-15 at 1 : 2 ratio). These results demonstrate that synergy between IL-12 and IL-15 is effective even in the presence of potent suppression by nTreg, and that synergy increases not only the frequency of CFSE^dim⁡^ cells, but also the number of cell divisions within dividing cells.

### 3.5. IFN-*γ* Is Not Required for CpG-Induced IL-12 Production

**Figure 5:**
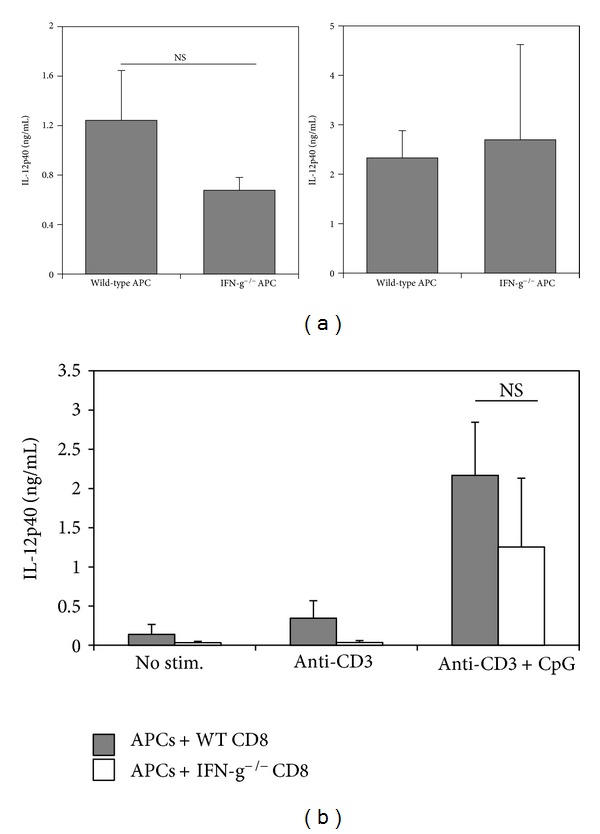
T cell-derived IFN-*γ* is not required for CpG-induced IL-12 production. (a) Thy1.2-depleted splenocytes were isolated from the spleens of naïve (left) or *T. cruzi*-infected (right) wild-type and *Ifng* ^−/−^ mice on day 9 p.i. Thy1.2-depleted splenocytes were then stimulated with CpG. After 72 hours, culture supernatants were harvested and tested for IL-12p40 by ELISA. (b) Wild-type (filled bars) or *Ifng* ^−/−^ (open bars) CD8^+^ T cells were purified from the spleens of *T. cruzi*-infected mice on day 9 p.i. and cocultured with wild-type Thy1.2-depleted APC for 72 hours in the presence of anti-CD3 or anti-CD3+CpG. Cell culture supernatants were then harvested and IL-12p40 levels were determined by ELISA. Results are representative of two independent experiments with two mice per group and are expressed as mean ± SD. NS: not significant.

Stimulation with CpG, as well as signals provided by memory CD8^+^ T cells such as IFN-*γ*, TNF-*α*, GM-CSF, and CD40L can cooperate in a synergistic fashion to promote IL-12p70 production [20]. For this reason, and because of the importance of IFN-*γ* to successful vaccine-induced immune responses, we wished to determine if IFN-*γ* was required for CpG-dependent upregulation of IL-12 by antigen-presenting cells. Thus, we stimulated Thy1.2-depleted splenocytes from naïve, wild-type, or *Ifng* ^−/−^ mice with CpG for 72 hours and tested supernatants for IL-12p40 protein. Although the levels of IL-12p40 were decreased in *Ifng* ^−/−^ cultures relative to wild-type cultures, there was no statistically significant difference between the groups (Figure 5(a), left). Additionally, Thy1.2-depleted splenocytes from *T. cruzi*-infected *Ifng* ^−/−^ mice stimulated with CpG produced as much IL-12p40 as wild-type splenocytes (Figure 5(a), right). These results suggest that APC-derived IFN-*γ* is not absolutely required for CpG-induced IL-12 production but may be necessary for optimal expression of IL-12. To further address the role of IFN-*γ* in CpG-induced IL-12 by APC, we set up cocultures consisting of Thy1.2-depleted splenocytes from wild-type uninfected mice with purified CD8^+^ T cells derived from either wild-type or *Ifng* ^−/−^ mice previously infected with *T. cruzi*. As expected, the addition of CpG to anti-CD3-stimulated cultures led to a strong induction of IL-12p40. Importantly, CpG-induced IL-12p40 production was not significantly affected when IFN-*γ*-deficient CD8^+^ T cells were utilized (Figure 5(b)). These results confirm that IFN-*γ* is not required for IL-12 production by APC in response to CpG stimulation.

### 3.6. The Synergistic Effects of IL-15 and CpG Are Dependent upon T-bet Expression

**Figure 6:**
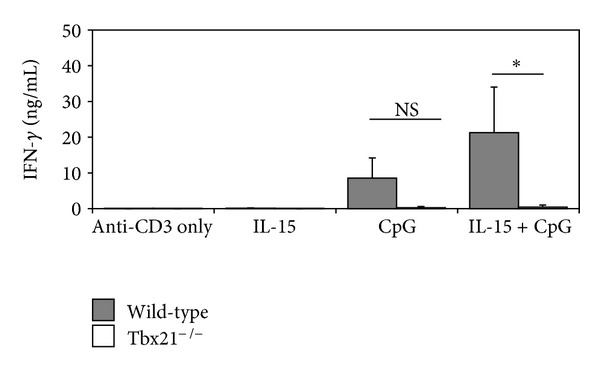
The combined effects of IL-15 and CpG on CD8^+^ T cell IFN-*γ* production require T-bet. Wild-type (filled bars) or *Tbx21* ^−/−^ (open bars) CD8^+^ T cells and Thy1.2-depleted APC were cocultured with anti-CD3 for 72 hours in the presence of IL-15, CpG, or IL-15+CpG. Cell culture supernatants were then harvested and IFN-*γ* levels were determined by ELISA. Results are representative of two independent experiments and are expressed as means ± SD. *P = 0.05; NS: not significant.

The T-box transcription factor T-bet (*Tbx21*) is critical for effector and memory CD8^+^ T-cell function, including IFN-*γ* production [21]. However, Eomes (*Eomesodermin*), a related transcription factor, can provide overlapping and redundant functions with T-bet [22]. Given that Eomes has the potential to compensate for T-bet functions, we wished to determine if the ability of IL-15 and CpG to enhance IFN-*γ* production by CD8^+^ T cells could be maintained in the absence of T-bet. Thus, CD8^+^ T cells from T-bet-deficient (*Tbx21* ^−/−^) mice were cocultured with APC in the presence of IL-15+CpG. Surprisingly, however, T-bet-deficiency in CD8^+^ T cells had a dramatic consequence, as the ability of IL-15+CpG to synergize and promote IFN-*γ* production was lost (Figure 6). These results demonstrate that synergy between IL-15 and CpG requires the expression of T-bet and suggests that Eomes cannot compensate for its absence.

## 4. Discussion

Because of their critical role in immunity to tumors, viruses, and intracellular parasites such as *T. cruzi *[13–16, 23–25], a major goal of vaccine development is to harness the potency of CD8^+^ T-cell responses. However, a challenge in developing CD8^+^ T-cell-based vaccines is the generation of potent effector T cells as well as long-term memory. One promising approach is the incorporation of adjuvants that act as TLR agonists and can augment CD8^+^ T-cell immunity. For example, synthetic CpG ODNs that express unmethylated CpG motifs have been the focus of much attention for their potential use as vaccine adjuvants due to their ability to promote type I immunity, including antigen-specific T- and B-cell responses [26]. Recently, common gamma-chain family cytokines such as IL-15 has also been considered as candidates for improving vaccine responses. While there are numerous studies showing the beneficial effects of CpG or IL-15, the potential synergy of these two immune modulators remains to be determined.

In this study, our goal was to determine if IL-15 and CpG could function in a synergistic manner to promote CD8^+^ T-cell responses. We show that CpG alone had the ability to enhance IFN-*γ* production by polyclonally activated CD8^+^ T cells. However, when CpG and IL-15 were used in combination, there was a profound and synergistic increase in IFN-*γ*. The synergistic effect required only low concentrations of IL-15 that otherwise could not directly induce IFN-*γ*. To expand upon this observation, we wished to determine if the synergistic effect could be recapitulated with antigen-specific CD8^+^ T cells. Splenocytes from *T. cruzi*-infected mice restimulated with Tskb20 peptide in the presence of both CpG and IL-15 showed an increase in IFN-*γ* production that was greater than with CpG alone or IL-15 alone. These findings support the notion that synergy between CpG and IL-15 can enhance antigen-specific CD8^+^ T-cell responses. Although synergy was less dramatic with T cells from *T. cruzi*-infected mice, we believe the difference is that Tskb20-specific T cells, which are generated *in vivo* in response to a natural infection, have differentiated under highly stimulatory conditions and may be refractory to further stimulation *ex vivo*. If true, it would suggest that the efficacy of CpG and IL-15 may be better suited for preventative vaccines as opposed to therapeutic vaccines. Nevertheless, synergy between CpG and IL-15 was evident when used to stimulate *T. cruzi*-specific CD8^+^ T cells in the absence of antigen.

Stimulation of APC with CpG promotes their maturation and increases the production of proinflammatory cytokines, especially IL-12, that favor the development of type I immunity [11, 26, 27]. Thus, we wanted to determine the role of IL-12 in CpG-mediated synergy with IL-15 on CD8^+^ T-cell function. Not surprisingly, we observed that T-cell-depleted splenocytes from naïve or *T. cruzi*-infected mice produced IL-12 in response to stimulation with CpG, whereas IL-15 had no effect on IL-12 production. Importantly, addition of IL-15 to CpG-stimulated cultures did not lead to a further increase in IL-12, suggesting that the synergy between CpG and IL-15 is not due to IL-15-mediated enhancement of IL-12 production. This observation is in slight contradiction to a recent study in which it was shown that IL-15 is important for CpG-induced IL-12 production by dendritic cells [19]. However, this discrepancy could be explained by the possibility that low levels of IL-15 were produced as a result of *T. cruzi* infection, making it such that additional treatment with IL-15 could not further enhance CpG-induced IL-12 production. To determine the importance of this IL-12, we observed that neutralization of IL-12 abrogated the synergistic effect on IFN-*γ* production by CD8^+^ T cells. Furthermore, antigen-specific CD8^+^ T cells stimulated in the presence of CpG and IL-15 produced significantly less IFN-*γ* when cultured with IL-12-deficient APC. Thus, our results demonstrate that the enhancement of CD8^+^ T-cell IFN-*γ* production provided by the combination of CpG and IL-15 requires CpG-induced IL-12.

In a previous study, it was reported that memory CD8^+^ T cells are required for APC to produce optimal levels of IL-12 in response to CpG stimulation [20]. Specifically, it was observed that IFN-*γ*, TNF-*α*, and GM-CSF produced by CD44^hi^ memory CD8^+^ T cells synergized with CpG to prime dendritic cells for further IL-12 production. For this reason, we examined whether T cell-derived IFN-*γ* was necessary for CpG-induced IL-12 production by APC. However, our results indicated that IFN-*γ*, regardless of the source, was not necessary for APC to produce IL-12 in response to CpG stimulation. Thus, although IFN-*γ* appeared to be dispensable in our study, it remains possible that TNF-*α* and/or GM-CSF from *T. cruzi*-specific memory CD8^+^ T cells could be priming APC for CpG-induced IL-12 production. Nevertheless, IL-12 is critical for the observed synergistic effects between CpG and IL-15. The findings we report here compliment those reported by Wysocka et al. [6], in which the beneficial effects of CpG and IL-15 were observed following the treatment of human NK and CD8^+^ T cells isolated from patients with cutaneous T-cell lymphoma. Even though analysis of CD8^+^ T cells was limited to upregulation of CD69 expression, CpG, and IL-15-augmented IFN-*γ* production by peripheral blood mononuclear cells. Numerous studies have shown that CpG can increase the immunogenicity of vaccines against cancer and infectious diseases. For instance, CpG can accelerate the development and enhance the magnitude of vaccine-induced immune responses. In the case of *T. cruzi* infection, CD8^+^ T cells are critical for host protection. However, their development is delayed in comparison to other viral and bacterial infections and this delay has been attributed to poor TLR stimulation. Indeed, CpG administration in combination with a TLR-2 agonist significantly accelerated the development of CD8^+^ T-cell responses to *T. cruzi* [28]. Thus, vaccine approaches against infectious agents will likely benefit greatly from the immunostimulatory properties of CpG.

With respect to the contribution of IL-15, we observed that IL-15 increases cell division among activated CD8^+^ T cells, as well as increases cell viability. This is not a trivial point. For example, local IL-15 production in the heart of *T. cruzi*-infected persons correlates with increased numbers of CD8^+^ T cells [29]. Furthermore, an important finding we report here is that IL-15 can synergize with IL-12, the main effector of CpG stimulation, to overcome potent suppression mediated by CD4^+^CD25^+^ regulatory T cells (nTreg). The combination of IL-15 and IL-12 provided a significant boost to both CD8^+^T-cell proliferation and IFN-*γ* production despite high numbers of Treg. Since Treg function has been reported to interfere with immune responses to *T. cruzi* [30, 31], this would be another advantage of using CpG and IL-15 to boost immunity to *T. cruzi*. We propose that the ability of IL-15 to promote CD8^+^ T-cell survival and cell division, as well as function in the presence of Treg-mediated suppression, contributes to its effectiveness in synergizing with CpG. It should be noted that although the effects described above for IL-15 impact CD8^+^ T cell numbers, it was recently reported that IL-15 may enhance expression of the low-affinity IL-12 receptor *β*1 in both mouse and humans [32, 33].

To identify the T-cell-intrinsic factors that are important for the reported effects of CpG and IL-15, we turned our attention to the transcription factors T-bet and Eomes. T-bet and Eomes are critical for CD8^+^ T-cell development and function [21]. For example, T-bet regulates the generation of antigen-specific CD8^+^ T cells [17] and promotes effector functions like IFN-*γ* production [34]. Likewise, Eomes performs similar functions such as promoting the production of cytotoxic effector molecules and IFN-*γ* [22]. Here, we demonstrate that T-bet is required for the synergistic enhancement of IFN-*γ* production by CpG and IL-15. Although Eomes can compensate for many of the functions of T-bet in CD8^+^ T cells [21], it did not appear to mediate the synergistic effect of CpG and IL-15 and their ability to enhance IFN-*γ* production. Therefore, T-bet expression in CD8^+^ T cells is critical for the beneficial effects of CpG and IL-15 treatment.

In summary, we propose that CpG causes APC to release IL-12, a powerful cytokine that serves as “Signal 3” for CD8^+^ T cells. However, the IL-12 cannot act unless T cells are activated and express the appropriate receptor to respond to IL-12. IL-15, in addition to its ability to promote CD8^+^ T-cell survival and proliferation, may enhance T-cell responsiveness to IL-12 and thus increase IFN-*γ*. We believe the synergy will enhance both immediate and long-term CD8^+^ T-cell responses. This has great potential in clinical trials, because CpG do not appear to have the same toxicity as treatment with exogenous IL-12, which has been reported in cancer patients despite positive therapeutic effects. In terms of IL-15, adoptive transfer of T cells into a lymphopenic tumor-bearing host (which typically occurs after whole body irradiation) leads to increased IL-15-dependent proliferation, expansion, and CD8^+^ T-cell effector function. The results of our study provide additional evidence of the potential efficacy of CpG and IL-15 and how it may improve vaccine approaches against cancer and infectious diseases. Not only do we demonstrate that CpG and IL-15 are more effective when used in combination, but our findings also provide new insight into the immune mechanisms responsible for the observed synergy between CpG and IL-15. Because of the considerable interest in TLR agonists as vaccine adjuvants, we believe our study will provide additional evidence that TLR agonists, when combined with common gamma chain cytokines, have the potential to dramatically enhance vaccine efficacy.
